# Trade-off between travel distance and prioritization of high-reward sites in traplining bumblebees

**DOI:** 10.1111/j.1365-2435.2011.01881.x

**Published:** 2011-12

**Authors:** Mathieu Lihoreau, Lars Chittka, Nigel E Raine, Gaku Kudo

**Affiliations:** Biological and Experimental Psychology Group, School of Biological and Chemical Sciences, Queen Mary University of LondonMile End Road, London E1 4NS, UK

**Keywords:** *Bombus terrestris*, distance reward trade-off, optimal foraging theory, spatial cognition, trapline foraging, Travelling Salesman Problem

## Abstract

**1.**Animals exploiting renewable resource patches are faced with complex multi-location routing problems. In many species, individuals visit foraging patches in predictable sequences called traplines. However, whether and how they optimize their routes remains poorly understood.

**2.**In this study, we demonstrate that traplining bumblebees (*Bombus terrestris*) make a trade-off between minimizing travel distance and prioritizing the most rewarding feeding locations.

**3.**Individual bees trained to forage on five artificial flowers of equal reward value selected the shortest possible route as a trapline. After introducing a single highly rewarding flower to the array, they re-adjusted their routes visiting the most rewarding flower first provided the departure distance from the shortest possible route remained small (18%). When routes optimizing the initial rate of reward intake were much longer (42%), bees prioritized short travel distances.

**4.**Under natural conditions, in which individual flowers vary in nectar productivity and replenish continuously, it might pay bees to prioritize highly rewarding locations, both to minimize the overall number of flowers to visit and to beat competitors.

**5.**We discuss how combined memories of location and quality of resource patches could allow bees and other traplining animals to optimize their routing decisions in heterogeneous environments.

## Introduction

Foraging (the activity of searching for, finding and consuming food) requires animals to make decisions whose outcomes can be crucial for their fitness (Stephens, Brown & Ydenberg 2007). According to foraging theory, individuals should develop strategies to maximize their net rate of energy intake per unit time, thus exploiting the most profitable resources in the least possible time (e.g. Fretwell & Lucas 1970; Charnov 1976). If the potential choices between food locations are already known by the individual and included in a foraging sequence, the optimization task becomes analogous to the well-known Travelling Salesman Problem (finding the shortest circuit to visit all locations in an array exactly once) for which no efficient general mathematical solution is yet known (Applegate *et al.* 2006). Central place foragers collecting patchily distributed resources that replenish over time are faced with such multi-location routing problems (Anderson 1983). In these species, individuals often repeat foraging circuits, visiting a particular set of patches in a predictable non-random order referred to as ‘trapline foraging’ [e.g. pollinating insects (Janzen 1971; Heinrich 1976), birds (Davies & Houston 1981; Gill 1988), bats (Lemke 1984; Racey & Swift 1985), primates (Di Fiore & Suarez 2007; Noser & Byrne 2010) and rodents (Reid & Reid 2005)]. Despite the taxonomically widespread nature of this behaviour, only a few studies have investigated whether and how traplining animals optimize their routing decisions. Primates (Menzel 1973; Cramer & Gallistel 1997) and bees (Lihoreau, Chittka & Raine 2010) tested under laboratory conditions have been shown to find the shortest possible route to visit multiple resource patches of identical reward value, thus solving simple forms of the Travelling Salesman Problem. However, these findings contrast with a number of field observations in which free-ranging animals travel long routes, often bypassing the nearest resource patches to visit more distant ones (Janzen 1971; Cunningham & Janson 2007; Noser & Byrne 2007). Such a discrepancy between laboratory and field studies suggests that traplining animals not only attempt to minimize travel distances, but might also use information about the quality of food patches to make foraging decisions, thus raising the important issue of how heterogeneity of resource value could affect potential solutions to multi-location routing problems.

**Fig. 1:**
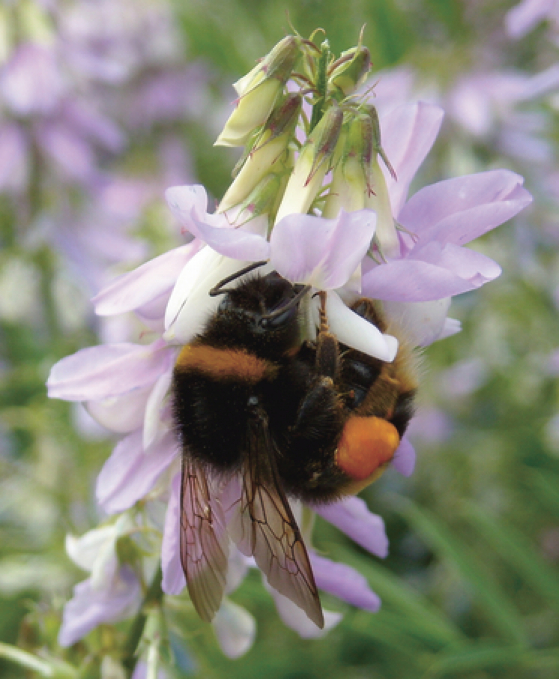
Bumblebee (*Bombus terrestris audax*) queen collecting legume pollen. Photograph by Nigel E. Raine.

In this study, we explore the possibility of a trade-off between travel distance and the prioritization of high-reward locations by traplining bumblebees (*Bombus terrestris*; Fig. 1). Recent evidence indicates that bees moving between distant feeding locations (or flower patches) of identical reward value reduce their overall flight distances after extensive exploration, often selecting the shortest possible route as a trapline (Lihoreau, Chittka & Raine 2010). A wealth of information also indicates that bees are highly sensitive to the quality of floral rewards and often show a preference for visiting flowers with the best rewards [e.g. pollen quantity (Cresswell & Robertson 1994), nectar warmth (Dyer *et al.* 2006), nectar quantity (Makino & Sakai 2007) and nectar concentration (Whitney *et al.* 2008)]. However, whether bees integrate information about both the value and location of flowers (or flower patches) to optimize their routing decisions remains unexplored. Under natural conditions, in which individual flowers vary in their patterns of nectar production and refill continuously, it might be advantageous to prioritize visits to highly rewarding flowers both to minimize the overall number of flowers that need to be visited to fill the bee's crop to capacity and to harvest large nectar rewards before competitors do.

To test this hypothesis, we observed bees developing traplines in an array of five artificial flowers. By manipulating flower location and the relative value of sucrose solution reward provided by each flower, we generated three experimental treatments providing increasing levels of discrepancy between the length of the routes minimizing overall flight distance (the shortest possible route) and the length of the routes maximizing the initial rate of reward intake (by visiting the most rewarding flower first).

## Materials and methods

**Fig. 2:**
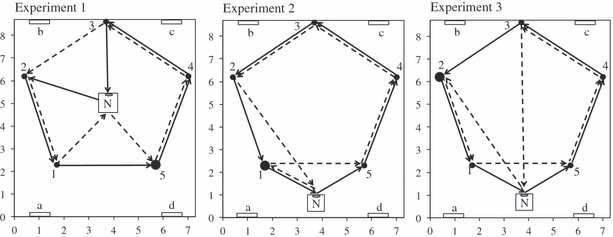
Spatial configurations of flowers. Black circles (1–5) indicate the location of flowers (small circles = low rewarding value; large circles = high rewarding value), N is the nest-box (grey ellipse = entrance) and white bars (a–d) the geometric poster landmarks. Black arrows show examples of anticlockwise routes minimizing travel distance (the shortest possible route). Dashed arrows show examples of anticlockwise routes maximizing initial rate of food intake by visiting the most rewarding flower first (assuming a constant directionality of movement). Experiment 1: a bee minimizing travel distance would always maximize its initial rate of reward intake. Experiment 2: a bee maximizing its initial rate of reward intake would fly a route 18% longer than a bee minimizing travel distance. Experiment 3: a bee maximizing its initial rate of reward intake would fly a route 42% longer than a bee minimizing travel distance. Scale is in metres. Cartesian coordinates of all objects in the flight room are given in Table S1.

Experiments were carried out in an indoor flight room (870 × 730 × 200 cm) set up in a greenhouse (temperature range: 15–20 °C; photoperiod: 12 h dark/12 h light). The windows of the greenhouse were obscured with white paint (Leyland, Bristol, UK) and controlled illumination was provided by high-frequency fluorescent lighting [TMS 24F lamps with HF-B 236 TLD (4·3 kHz) ballasts (Philips, Eindhoven, The Netherlands) fitted with Activa daylight fluorescent tubes (Osram, Munich, Germany)], which simulates natural daylight above the bee flicker fusion frequency. Subjects were workers from a commercially obtained *B. terrestris* colony (Syngenta Bioline Bees, Weert, The Netherlands), housed in a wooden nest-box. Movements of bees from the nest-box to the flight room were carefully controlled using shutters in the transparent entrance tube fitted on one side of the nest-box (Fig. 2). Bees were marked with individually numbered tags within 1 day of emergence from pupae. The colony was provided with *ad libitum* pollen and workers collected sucrose solution (40% w/w) from remote-controlled artificial flowers in the flight room (see Fig. S1 in Supporting Information). Each flower was placed on a wooden support (height 50 cm). To help bees navigate accurately, four geometric patterned posters (height = 120 cm; width = 85 cm) were fixed to the walls in each corner of the room as landmarks (Fig. 2, for details see Fig. S2).

### Training

Bees were allowed to forage freely on five flowers arranged in a linear patch (distance between neighbouring flowers = 10 cm) placed 1 m in front of the nest-box entrance (perpendicular to the entrance tube). Feeding cups of the flowers were refilled *ad libitum* (5 μL of sucrose per flower). Regular foragers that made at least five foraging bouts (collected sucrose from the flowers until they filled their crop and returned to the nest to deposit their nectar load) in two hours were selected for testing [(*n*=15 bees; age: mean = 23·93 ± 3·44 days (SE); thorax width: mean = 5·05 ± 0·08 mm (SE)]. The average volume of sucrose ingested by each of these bees during three additional foraging bouts was used to estimate their individual crop capacity (range: 120–190 μL).

### Tests

Bees were observed individually during 80 successive bouts on the same day, foraging on the five flowers arranged in a regular pentagon (distance range between flowers: 410–663 cm; Fig. 2; Table S1). As *B. terrestris* workers can visually detect a target that subtends a 3° angle (Kapustjansky, Chittka & Spaethe 2010), it is likely that the bees could detect all flowers from any location within the array (the minimum angle that subtends a flower of 60 cm high within the dimensions of the room is 3·95°). The volume of sucrose solution provided by each flower was adjusted for each test bee so that they had to visit all five flowers during a foraging bout to fill their crop. Feeding cups of flowers were refilled only after each foraging bout was completed.

During phase 1 of observations (the first 40 foraging bouts), all five flowers delivered identical volumes of sucrose solution (reward volume = 1/5th of the crop capacity). Thus, the bee could develop a route based only on the spatial distribution of flower locations. During phase 2 of observations (the last 40 foraging bouts), one flower delivered six times as much sucrose solution as the other four flowers (reward volume of the most rewarding flower = 3/5th of the crop capacity; reward volume of the four other flowers = 1/10th of the crop capacity). Hence, the bee could use both the location of flowers and the relative value of rewards they provide to adjust its route. If the bee visits the highly rewarding flower first, its initial rate of reward intake is improved. However, the total volume of sucrose solution obtained by visiting all five flowers in a single bout was always identical.

We conducted three experiments (*n*=5 bees per experiment) that provided increasing levels of discrepancy between the length of the route minimizing travel distance (the shortest possible route) and the length of the route maximizing the initial rate of reward intake (the route starting with the most rewarding flower) during phase 2 (Fig. 2; Tables S1 and S2). Based on the observation that individual bees are highly consistent in their tendency to fly around the flower array in a clockwise or anticlockwise direction when a route is established (Cheverton 1982; Lihoreau, Chittka & Raine 2010), the location of the most rewarding flower during phase 2 was adjusted for each bee to generate the highest level of discrepancy between routes minimizing travel distance and maximizing initial rate of reward intake. For instance, increasing the rewards provided by flower 1 for a bee turning anticlockwise (sequence: 54321) would force that bee to make a detour from the shortest possible route to optimize its initial rate of reward intake (sequence: 15432).

Experiment 1: The nest-box entrance was placed at the centre of the pentagon (equidistant from all flowers) so that a bee visiting all five flowers could always travel the same distance (the shortest possible route) irrespective of which flower was visited first or its directionality of movements (clockwise or anticlockwise). In phase 2, the most rewarding flower was selected at random. A bee maximizing its initial rate of reward intake would also minimize travel distance, assuming it was consistent in its directionality of movements between phases 1 and 2.
Experiment 2: The nest-box was placed outside the pentagon (equidistant between flowers 1 and 5) so that a bee visiting all five flowers with a given directionality of movements would travel different distances in relation to the first flower visited. The most rewarding flower in phase 2 was selected as the flower visited fifth by the test bee during phase 1 (either flower 1 or 5). A bee maximizing its initial rate of reward intake would fly a route 18% longer than a bee minimizing travel distance, assuming it was consistent in its directionality of movements between phases 1 and 2.
Experiment 3: As in experiment 2, the nest-box was placed outside the pentagon (equidistant between flowers 1 and 5), so that a bee visiting all five flowers with a given directionality of movements would travel different distances in relation to the first visited flower. The most rewarding flower in phase 2 was the one the bee visited most often in fourth position during phase 1 (either flower 2 or 4). A bee maximizing its initial rate of reward intake would fly a route 42% longer than a bee minimizing travel distance, assuming it was consistent in its directionality of movements between phases 1 and 2.

We recorded the time at which the bee left/entered the nest-box and visited each flower. The total time spent flying per foraging bout was calculated by subtracting the time spent landed on each flower from the bout duration. The total distance flown by the bee was estimated as the minimum distance flown in a straight line between flower visits and flights to/from the nest. Between testing bees, we cleaned the landing platform of each flower with ethanol solution (70% w/w). Tested bees were freeze-killed and measured (thorax width).

### Data Analysis

Data were analysed using R statistical software 2.10.1 (R Development Core Team 2009). All means are given with standard errors, and normality of data was assessed using Shapiro-Wilk tests. We excluded from analyses foraging bouts in which the bees did not visit all five flowers (7·27 ± 0·73 bouts per bee, *n*=15). Most of these bouts were observed in naïve bees (>56% in the first 20 bouts of phase 1) and equally distributed among individuals (Chi-square test, *χ*^2^_14_ = 15·54, *P*=0·342; Fig. S3).

We investigated the effect of experience (cumulative number of foraging bouts) on the foraging performance of bees (flight duration, number of flower visits, flight distance), by analysing complete flower visitation sequences (including all revisits to the same flower) with Generalized Linear Mixed Models (GLMMs). We explored the effect of nectar load (cumulative volume of sucrose solution collected during the foraging bout) on flight speed (shortest flight distance between two successive flower visits divided by flight duration) using a similar procedure. In all models, identity and body size of bees were included as random effects.

To investigate the spatial geometry of routes, we used only the first visit to each flower, thus excluding revisits to empty flowers as described in Lihoreau, Chittka & Raine (2010). Bees decreased drastically their frequency of revisits per foraging bout with experience, from an average of 3·41 ± 0·36 revisits in the first 10 foraging bouts of phase 1 to 0·42 ± 0·22 in the last 10 foraging bouts of phase 2 (GLMM: experience effect, *t*_1051_ = −13·18, *P*<0·001). Most of these revisits by inexperienced bees were immediate returns to the flower just visited (68·51% of all revisits, *n*=3474), rather than returns to different flower locations and were therefore uninformative with respect to the core structure of routes. As there are 120 possible routes to visit five flowers once (5!), we used multinomial tests (random probability = 1/120) to analyse the frequency of route usage by each individual bee. Thus, routes used at least twice by a bee during phase 1 or 2 were used significantly more often than expected by chance (*P*<0·05). We compared the frequencies of route usage among experiments (number of different routes, number of shortest possible routes, number of clockwise or anticlockwise routes used) using GLMs. We defined the ‘trapline’ as the route used most frequently by a bee after training, during the 20 last foraging bouts of each phase.

To evaluate the variability in the spatial geometry of routes, for each individual bee, we calculated a similarity index (SI) for pairs of flower visitation sequences observed the most frequently. This procedure takes into account insertions, deletions and substitutions to any primary sequence and allows us to identify changes between two routes starting and ending at the nest (Thomson, Slatkin & Thomson 1997; Lihoreau, Chittka & Raine 2010). SI ranges between 0 (the two visitation sequences are completely different) and 1 (the two visitation sequences are identical). Because each bee used at least three routes more often than expected by chance in phases 1 and 2 of experiments (Fig. S3), we calculated three SI values per bee (one for each pairwise comparison between the three most often used visitation sequences) and analysed the average SI value. To determine whether these routes were significantly more similar than expected by chance, we generated 300 visitation sequences to the five flowers using a pseudo-random algorithm (the bee must visit the five flowers once without revisits) and calculated the mean SI values for bins of three pairs of routes. This allowed us to compare the 15 average SI values from our observations to 100 average SI values obtained from our null model using a *t*-test.

We investigated consistency in the directionality of movements by comparing the number of bouts in which bees visited the flowers in a clockwise or anticlockwise sequence, irrespectively of the first flower visited. For each bee, we calculated a directionality index (DI) by subtracting the number of clockwise sequences from the number of anticlockwise sequences, during phase 1 and phase 2. A negative DI indicates a tendency for the bee to turn clockwise, while a positive DI indicates a tendency to turn anticlockwise. Significance of directionality biases was assessed by comparing the frequency of clockwise and anticlockwise sequences using binomial tests (random probability 0·5). We compared DI values between phases 1 and 2 using a Wilcoxon test.

## Results

### Phase 1: Equally Rewarding Flowers

Bees tested in the array of five equally rewarding flowers (Fig. 2) improved their foraging performance with experience by reducing their total flight duration (GLMM: experience effect, *t*_525_ = −11·38, *P*<0·001), their number of revisits to empty flowers (GLMM: experience effect, *t*_525_ = −2·83, *P*=0·005) and their flight distance (GLMM: experience effect, *t*_525_ = −4·14, *P*<0·001) per foraging bout. Flight speed did not vary significantly in relation to the cumulative volume of sucrose solution collected during the foraging bout (GLMM: nectar load effect, *t*_3244_ = −1·17, *P*=0·241).

**Fig. 3:**
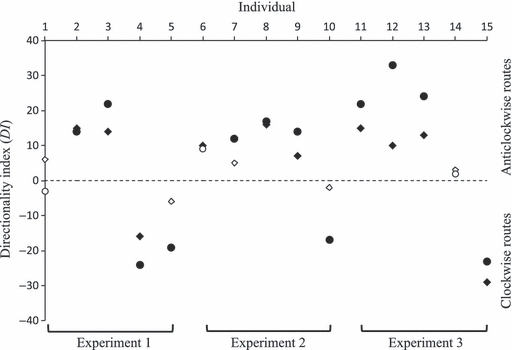
Directionality of bee movement between flowers. Plotting symbols represent values of directionality indices (DI) for each bee during phase 1 (circles) and phase 2 (diamonds) of observations. Negative DI values indicate a tendency for the bees to follow a clockwise route between flowers. Positive DI values indicate a tendency to move in an anticlockwise direction. Symbols in black illustrate significant biases in directionality compared with a null hypothesis that clockwise and anticlockwise routes are equally likely (binomial test with a random probability 0·5: *P*<0·05).

Detailed analyses of flower visitation sequences (excluding revisits to empty flowers) indicate that each bee showed a strong tendency to visit the five flowers in either a clockwise or an anticlockwise sequence irrespective of the flower they chose to visit first (Fig. 3). This directionality of movements was significantly biased for 10 of the 15 bees tested (binomial test: *P*<0·05). On average, the bees used 6·07 ± 0·58 routes (*n*=15) more often than expected by chance (multinomial test: *P*<0·05) and repeated three of these routes in 48·84 ± 5·53% (*n*=15) of all their foraging bouts (Fig. S3). These three most frequently used routes were significantly more similar to each other than expected by chance (SI observed routes: 0·52 ± 0·04, SI random routes: 0·43 ± 0·01; *t-*test: *t*_15·868_ = 2·88, *P*=0·011), indicating that each bee was using flower visitation sequences with only minor variations in most of their foraging bouts.

**Fig. 4:**
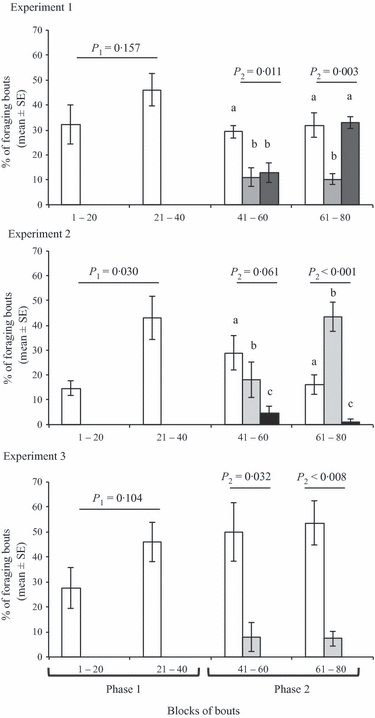
Frequency of route usage. In phases 1 and 2 of experiments, white columns represent the mean percentage of foraging bouts in which bees followed routes that minimized travel distance in relation to the cumulative number of foraging bouts completed (blocks of 20 successive bouts). In phase 2, grey columns represent the mean percentage of bouts in which bees followed routes that maximized initial rate of food intake and black columns the mean percentage of bouts in which bees followed routes that optimized both (minimized travel distance and maximized initial rate of food intake). *P*_1_: Generalized Linear Mixed Model (GLMM) with identity-link function (categorical variable: foraging bouts, random factor: individual). *P*_2_: GLMM with identity-link function (categorical variable: type of route, random factor: individual). Different letters (a, b, c) above columns indicate significant differences between percentages of foraging bouts within blocks of 20 bouts (*t*-tests).

The bees flew one of the shortest possible routes in 36·99 ± 3·49% (*n*=15) of their 40 foraging bouts (Fig. S3). This tendency to use one of the shortest possible routes increased between the first and last 20 foraging bouts (Fig. 4). During the last 20 bouts, all bees used one of the shortest possible routes as a trapline (the route they used most often: 25·19 ± 4·19% of the foraging bouts, *n*=15), by moving either clockwise (four bees) or anticlockwise (11 bees) between flowers. Thus, bees foraging on five equally rewarding flowers gradually minimized their overall travel distances as they gained experience of the array irrespective of the nest location relative to the flower array. We found no significant difference between bees in the three experimental treatments (GLMs: number of routes used; *χ*^2^_2_ = 1·66, *P*=0·436; usage frequency of the shortest possible route, *F*_2,12_ = 0·82, *P*=0·465; directionality of traplines, *χ*^2^_2_ = 0·66, *P*=0·719).

### Phase 2: Unequally Rewarding Flowers

After the reward values of all five flowers were changed (Fig. 2), bees continued to improve their overall foraging performance. As they built up experience with the flower array, bees followed shorter routes (GLMM: experience effect, *t*_557_ = −2·14, *P*<0·016) and made fewer revisits to empty flowers (GLMM: experience effect, *t*_557_ = −2·30, *P*=0·022). However, they did not reduce the time they spent in flight during each bout (GLMM: experience effect, *t*_557_ = −0·64, *P*=0·521). Like in phase 1, flight speed was not significantly affected by the amount of sucrose solution collected (GLMM: nectar load effect, *t*_1636_ = 1·64, *P*=0·101).

All but one bee continued to follow the same directionality of movements they exhibited in phase 1, visiting the flowers in either a clockwise or an anticlockwise sequence (Fig. 3). Directionality of movements was significantly less pronounced in phase 2 than in phase 1 (DI phase 1: 11·07 ± 1·77, DI phase 2: 17 ± 2·15; Wilcoxon test: *V*=92, *P*=0·014, *n*=15), indicating that bees explored new solutions in the presence of the highly rewarding flower. As in phase 1, each bee used on average 6·13 ± 0·42 routes (*n*=15) more often than expected by chance (Fig. S3). The three routes they followed the most frequently were used in 56·61 ± 5·92% of bouts and were significantly more similar to each other than expected by chance (SI observed routes: 0·50 ± 0·03, SI: random routes: 0·43 ± 0·01; *t-*test: *t*_16·008_ = 2·19, *P*=0·043).

**Fig. 5:**
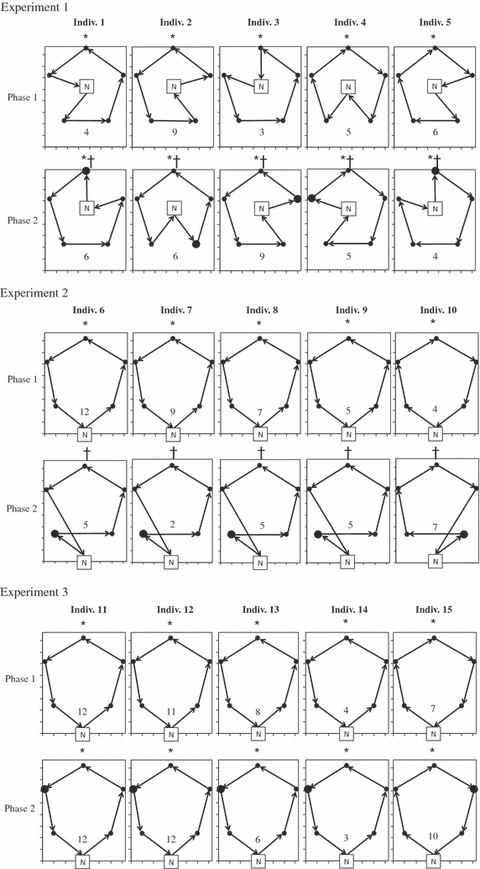
Spatial geometry of routes. For each bee, diagrams represent the geometry of the most frequently used route (trapline) in the last 20 foraging bouts of phase 1 (i.e. bouts 21–40: upper rows) and phase 2 (i.e. bouts 61–80: lower rows) of experiments. Black circles indicate the spatial location of flowers (small circles = low rewarding value; large circles = high rewarding value), N is the nest-box and arrows the direction of bee movements. Numbers are the frequency with which the route was observed during the last 20 foraging bouts of each phase of experiments. *Routes minimizing travel distances (phases 1 and 2). †Routes maximizing initial rate of food intake (phase 2). Scale is in metres.

During the first 20 foraging bouts, all bees flew one of the shortest possible routes significantly more often than a route starting with the most rewarding flower (Fig. 4). However, in the last 20 foraging bouts, this tendency to use one of the shortest possible routes varied greatly in relation to the magnitude of discrepancy between the length of the routes minimizing travel distance and those maximizing initial rate of reward intake. In the absence of a discrepancy in route length between minimizing travel distance and maximizing initial rate of reward intake (experiment 1), the bees used one of the shortest possible routes in 64·91 ± 4·34% (*n*=5) of their foraging bouts (Fig. 4). All five bees selected the shortest route starting with the most rewarding flower as their trapline (used in 30 ± 4·18% of foraging bouts, *n*=5), thus optimizing both travel distance and initial rate of reward intake (Fig. 5).

In the case of a small discrepancy in route length between minimizing travel distance and maximizing initial rate of reward intake (experiment 2), bees flew a long route starting with the most rewarding flower significantly more often than one of the shortest possible routes (Fig. 4). All five bees used one of these longer routes as their phase 2 trapline (used in 24 ± 4% of the foraging bouts, *n*=5), thus maximizing initial rate of reward intake rather than minimizing distance travelled (Fig. 5).

Finally, in the case of a large discrepancy in route length between minimizing travel distance and maximizing initial rate of reward intake (experiment 3), bees used one of the shortest possible routes significantly more often than a longer route starting with the most rewarding flower (Fig. 4). In contrast with results from experiments 1 and 2, bees remained consistent and used the same trapline in both phase 1 and 2 (in 43 ± 8·89% of foraging bouts, *n*=5), thus optimizing overall travel distances rather than initial rate of reward intake (Fig. 5). Altogether, these results show that bees attempted to optimize their routes by balancing the costs of flying long distances with the benefits prioritizing the most rewarding resources early in their trip.

## Discussion

In this study, we demonstrate that bumblebees make a trade-off between minimizing travel distance and prioritizing high-reward sites when developing multi-location routes. We discuss the adaptive value of this foraging strategy in natural conditions and the potential navigation mechanisms involved.

It has long been assumed that foragers exploiting patchily distributed resources should minimize their travel distances while maximizing the energy gained from food (Heinrich 1979; Pyke 1984). Even though many animal taxa have been shown to develop traplines (Janzen 1971; Heinrich 1976; Davies & Houston 1981; Lemke 1984; Racey & Swift 1985; Gill 1988; Reid & Reid 2005; Di Fiore & Suarez 2007; Noser & Byrne 2010), the optimization processes underpinning these routing decisions remain poorly understood. To our knowledge, this study is the first to explore the optimization performance of traplining animals in the presence of resources that differ in profitability. By manipulating the reward values of flowers within a stable spatial array, we provide evidence that bees attempt to optimize both travel distance and initial rate of reward intake. Conflicting situations, in which the two optimization processes force individuals to choose between different routes, indicate that bees use detours to visit the most rewarding flower first, as long as departure distance from the shortest possible route remains low. While prioritizing the most rewarding flowers might not have increased bees’ foraging efficiency in our laboratory controlled conditions, because they had to visit all flowers before filling their crops, it seems very likely that this strategy would be beneficial under natural conditions. In the wild, when flowers vary in reward value and replenish continuously, prioritizing the most rewarding flowers might allow bees to minimize the number of locations they need to visit before filling their crop to capacity, thus minimizing overall costs of travel associated with extended periods of flight and costs of carrying large nectar loads. Concomitantly, visiting the most rewarding flowers first might increase the competitiveness of traplining bees by increasing the probability to harvest large rewards before competitors (Paton & Carpenter 1984; Ohashi & Thomson 2005). In our experiments, the fact that the bees stopped prioritizing highly rewarding flowers for detour distances exceeding a critical value (between 4 and 9·5 m) and retained the shortest route as their trapline suggests that costs of flying long distances (Ellington, Machin & Casey 1990) and carrying nectar loads (Heinrich 1979) are the main factors determining the geometry of traplines at large spatial scales.

Our study not only demonstrates that bees trade-off reward and travel distance, but also provides new insights as to how they might optimize routes. Because there is no efficient mathematical method that provides general solutions to multi-location routing problems analogous to the Travelling Salesman Problem, traplining animals are often assumed to develop reasonably short routes using simple movement rules (heuristics): for instance, visiting the resources in their original discovery order (Janzen 1971), moving to the nearest available unvisited resource (or clusters of resources) until all resource locations have been visited (Bures, Buresova & Nerad 1992; Cramer & Gallistel 1997; Saleh & Chittka 2007) or making short movements after encountering rich resources and travelling longer distances after receiving poor resources (Keasar, Shmida & Motro 1996). In a recent study, we showed that bees navigating between distinct feeding locations do not exclusively rely on such heuristics, but are able to refine their routes after extensive exploration of their environment (Lihoreau, Chittka & Raine 2010), possibly using odometric information to compare overall distance flown (Srinivasan *et al.* 2000). The novelty of the present study is that bees also gradually adjust their routes in relation to changes in the reward value of the flowers they visit, suggesting that they acquire a combined memory of the location and quality of multiple food patches (Greggers & Menzel 1993). How bees encode and process both types of information still needs to be clarified. Like other traplining animals, bees have often been suggested to develop a ‘topological’ representation of space rather than encoding Euclidian relationships between environmental features (Collett, Fry & Wehner 1993; Di Fiore & Suarez 2007; Janson 2007), thus encoding spatial information using the relative position of landmarks and other salient features in their environment as a large number of path segments (vectors) grouped together to form a network of familiar routes (Dolins & Mitchell 2010). Under this hypothesis, a forager may be able to deviate from its established routes, but the potential for route innovation should be partially constrained by interference with learned associations/instructions that may not be easy to ignore or replace. We believe that our study provides evidence for such limitation. This is perhaps best illustrated with the results of experiment 2, in which bees had the opportunity to optimize both travel distance and initial rate of reward intake during phase 2 providing they could completely reverse the order in which they visited the flowers when compared with their original established trapline (e.g. change from 12345 to 54321). The fact that none of the bees achieved this, but instead remained highly consistent in their directionality of movements, suggests a limitation of their optimization abilities. In accordance with the topological hypothesis, a complete reversal of the foraging sequence may be difficult, as it implies learning a new flight vector to join each pair of flowers in the array. Conversely, the observed optimization pattern (despite being imperfect) constitutes a more parsimonious solution, as it implies a minimal disruption to the pre-existing trapline.

In conclusion, our study highlights the importance of resource heterogeneity in the routing decisions made by traplining animals, thus clarifying the discrepancy between laboratory observations where foragers have been described to use short routes between identical resource patches and field observations where they follow seemingly suboptimal circuits to join distant but probably highly productive resource patches. These observations highlight the need for further analyses of the role of the ecological factors (resource distribution, resource heterogeneity, social attraction and competition) in constraining routing decisions by traplining animals to refine our understanding of a taxonomically widespread foraging strategy.

## Supporting Information

**Figure. S1.** Artificial flower design.

**Figure. S2.** Appearance of the geometric patterned posters used as landmarks.

**Figure. S3.** Flower visitation sequences.

**Table S1.** Cartesian coordinates of the nest-box, the artificial flowers and the landmarks in the flight room.

**Table S2.** Flight distances according to flower visitation sequence.
