# Supplementary material for: Trade-off between travel distance and prioritization of high-reward sites in traplining bumblebees
Source: Funct Ecol. 2011 Dec;25(6):1284–92. doi: 10.1111/j.1365-2435.2011.01881.x (PMC3260656; doi:10.1111/j.1365-2435.2011.01881.x)
Supplement: Supplementary file 1 [file fec0025-1284-SD1.pdf]

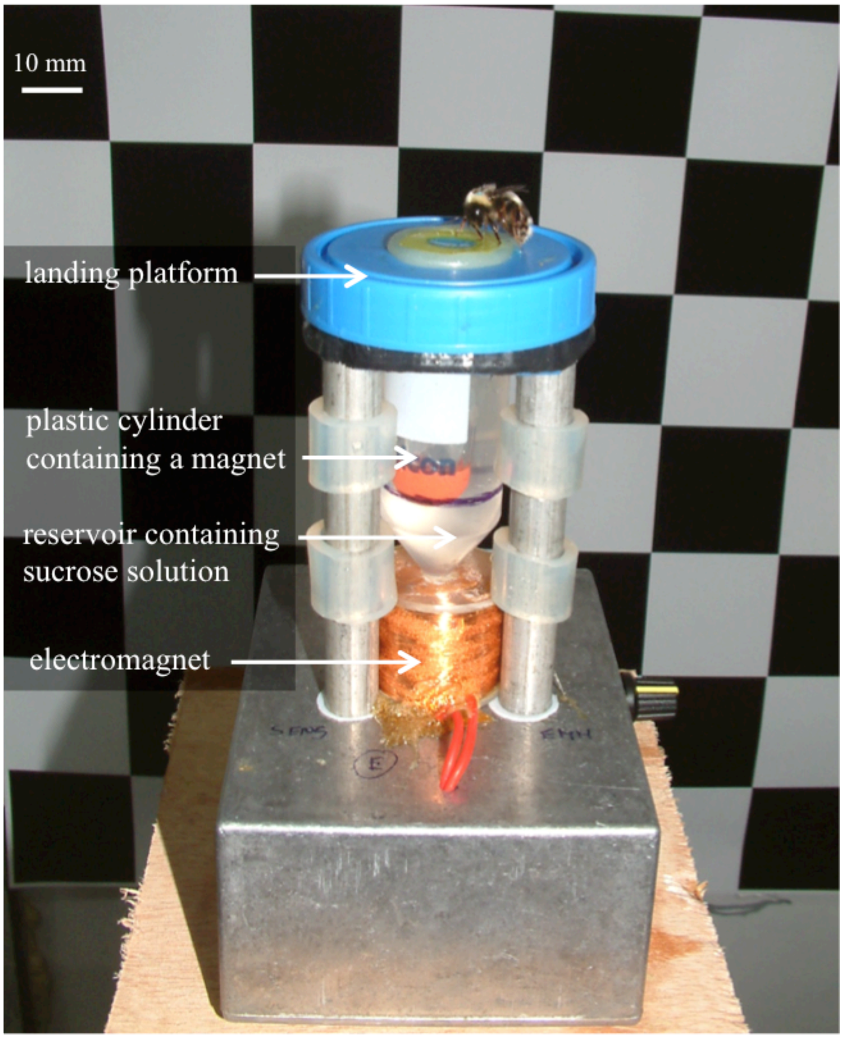

Fig. S1. Artificial flower design. Flowers were identical to those used by Lihoreau, Chittka & Raine (2010, *American Naturalist*, **176,**744-757). Bees alighting on the blue landing platform (plastic disk -Ø = 60 mm; height = 10 mm) can insert their proboscis through a narrow hole (Ø = 1 mm) in the centre and feed on sucrose solution from a feeding cup beneath (range: 5-30 µL, depending of the crop capacity of each test bee). The feeding cup is mounted on top of a plastic cylinder (Ø = 15 mm; height = 40 mm) containing a strong neodymium (NIB) magnet and floating in a vertical reservoir (Ø = 30 mm; height = 70 mm; volume = 50 mL) containing sucrose solution (40% w/w). Activation of the electromagnet beneath the reservoir pulls the float down below the surface of the sucrose solution, which acts to refill the cup. When the electromagnet is switched off, the float bobs up and the cup containing sucrose is guided into position by means of a plastic sleeve just below the feeding hole. The electromagnetic mechanism can be activated from a remote control box. These flowers can be accessed by a bee equally well when approached from all angles – so the direction from which they arrive or depart each flower is not constrained.
