# Supplementary material for: Trade-off between travel distance and prioritization of high-reward sites in traplining bumblebees
Source: Funct Ecol. 2011 Dec;25(6):1284–92. doi: 10.1111/j.1365-2435.2011.01881.x (PMC3260656; doi:10.1111/j.1365-2435.2011.01881.x)
Supplement: Supplementary file 2 [file fec0025-1284-SD2.pdf]

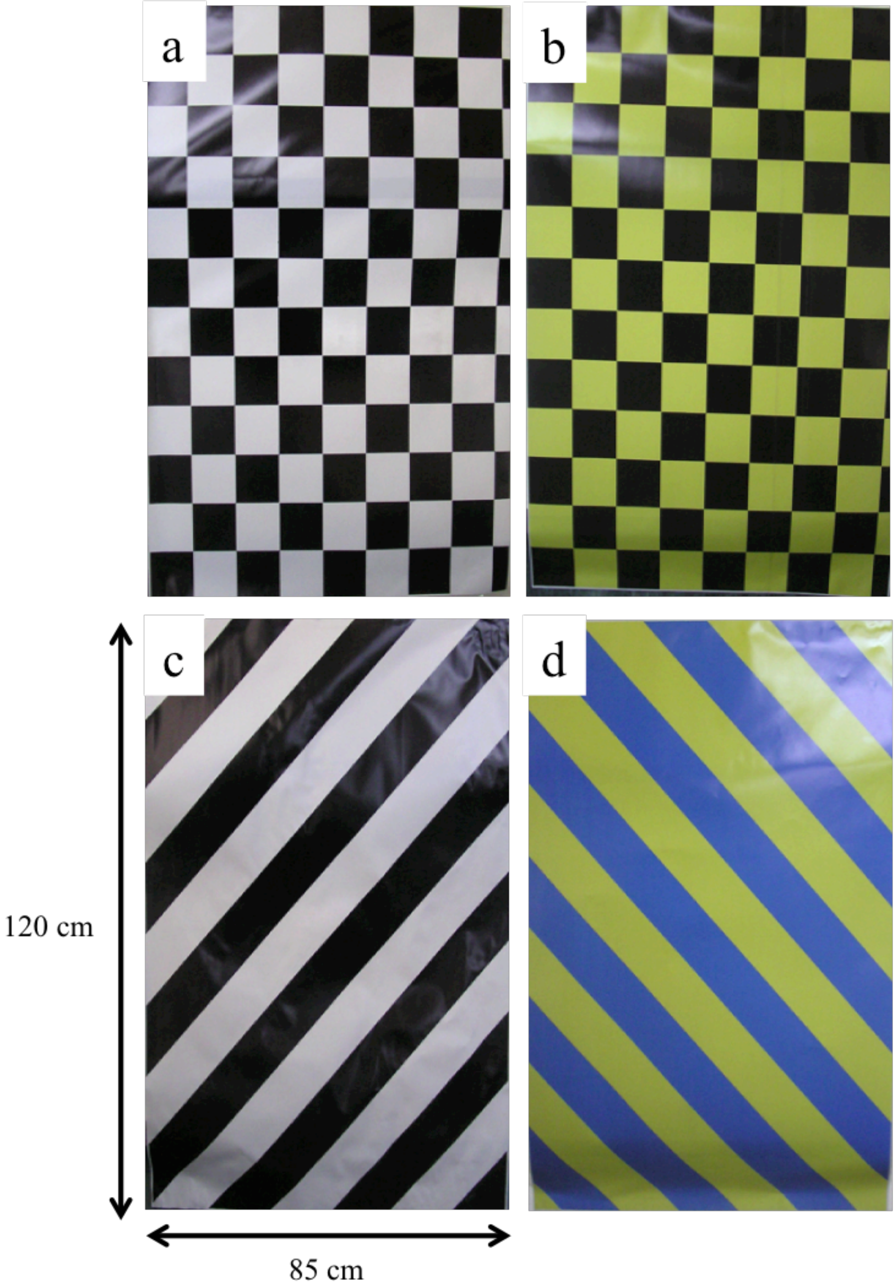

Fig. S2. Appearance of the geometric patterned posters (a-d) used as landmarks. Posters were fixed to the walls in each corner of the flight cage (Fig. 1).
