# Supplementary material for: Trade-off between travel distance and prioritization of high-reward sites in traplining bumblebees
Source: Funct Ecol. 2011 Dec;25(6):1284–92. doi: 10.1111/j.1365-2435.2011.01881.x (PMC3260656; doi:10.1111/j.1365-2435.2011.01881.x)
Supplement: Supplementary file 3 [file fec0025-1284-SD3.pdf]

Phase	Bout	Experiment 1					Experiment 2					Experiment 3				
		**indiv. 1**	**indiv. 2**	**indiv. 3**	**indiv. 4**	**indiv. 5**	**indiv. 6**	**indiv. 7**	**indiv. 8**	**indiv. 9**	**indiv. 10**	**indiv. 11**	**indiv. 12**	**indiv. 13**	**indiv. 14**	**indiv. 15**
Phase 1	bout 1	52143 †	1345	231	54123 †	321	54123 †	1354	35412	152	53142	514	32	124	54132	21345
	bout 2	2541	23514 †	12345 * †	13542 †	342	54213	45132	15432	12345 *	15342	123	1532	42315	45123	12354
	bout 3	45213 †	125	35412 †	15432 †	42153 †	54312	4351	34521	14523	51234	32154	53	45132	54321 *	51234
	bout 4	12453 †	153	35412 †	15234 †	45123 * †	12534	54321 *	32154	32451	5214	54123	1345	5413	43512	12345 *
	bout 5	5412	43125 †	21345 †	12543 †	45123 * †	54123	415	54312	51243	54213	43125	1323	54	12453	12345 *
	bout 6	21543 * †	32154 * †	54231 †	3421	54123 †	54312	51432	34152	54132	1523	51423	432	152	45123	12345 *
	bout 7	24513 †	12354 †	15432 †	51423 †	4325	54132	32415	54321 *	54123	21543	514	3125	23145	21543	51234
	bout 8	54213 †	35214 †	23154 †	14352 †	32154 * †	125	51342	41352	54321 *	14532	15432	3125	24153	43521	12345 *
	bout 9	51243 †	31254 †	23154 †	42135 †	4512	54321 *	23154	23415	51423	1513	54321 *	53421	54123	514	12345 *
	bout 10	21543 * †	34152 †	15423 †	54123 †	12435 †	54312	51234	21543	51432	15432	34521	1513	54	54123	12345 *
	bout 11	12	23154 †	21534 †	3425	25413 †	54321 *	25413	34251	54123	15432	1345	3121	123	123	54123
	bout 12	12354 †	43215 * †	21543 * †	21534 †	34215 †	125	15324	15432	54123	2314	34512	54321 *	25314	45312	23451
	bout 13	43215 * †	54312 †	5412	51432 †	51234 * †	12543	51234	12543	15423	23415	54312	54321 *	32145	45231	51234
	bout 14	43215 * †	453	54123 †	45123 * †	25413 †	512	32145	25143	32154	213	54321 *	54321 *	34125	43215	12345 *
	bout 15	21345 †	23154 †	2541	12345 * †	45123 * †	12345 *	45312	54321 *	21453	2341	132	54321 *	15234	21345	154
	bout 16	15423 †	51432 †	5412	51423 †	12345 * ††	54312	15234	34215	43215	21345	54312	5315	23154	45123	5421
	bout 17	23154 †	21543 * †	2341	51243 †	23451 * †	43512	51342	51243	21543	12345 *	54321 *	51234	34251	12345 *	12345 *
	bout 18	54321 * †	325	54321 * †	34512 * †	45123 * †	54312	121	54321 *	54123	15	12345 *	12354	15342	45123	25134
	bout 19	12543 †	54321 * †	54123 †	12543 †	51234 * †	15432	51324	32154	21543	54321 *	123	15432	54321 *	45231	45123
	bout 20	21345 †	51423 †	34152 †	21543 * †	51234 * †	12543	15324	12543	54123	12345 *	54321 *	15432	15342	21543	142
	bout 21	54321 * †	34251 †	21543 * †	52341 †	45123 * †	12354	43152	51342	51243	12345 *	12345 *	51432	53214	54312	5124
	bout 22	32154 * †	3542	21543 * †	23154 †	451	54321 *	51324	54321 *	51423	13452	15342	15243	54321 *	45123	34
	bout 23	12543 †	341	23154 †	51432 †	54312 †	54321 *	52341	54321 *	12543	23451	12345 *	51432	31245	45123	34512
	bout 24	21543 * †	34521 †	21354 †	21354 †	23154 †	12345 *	51234	12345 *	12354	12354	12543	54321 *	54321 *	52314	125
	bout 25	12345 * †	51234 * †	21543 * †	54312 †	45123 * †	12345 *	23145	51432	54132	21534	3125	51243	54321 *	31254	12345 *
	bout 26	32154 * †	435	23154 †	51423 †	23541 †	12345 *	54321 *	54123	52143	51432	54321 *	12345	23415	21354	23451
	bout 27	43215 * †	1534	21543 * †	12354 †	51234 * †	54321 *	54321 *	12354	24315	54123	54321 *	54321 *	32154	12354	12345 *
	bout 28	12345 * †	21543 * †	21534 †	54132 †	34512 * †	12345 *	51234	54321 *	15234	52341	54321 *	54321 *	15432	54123	12345 *
	bout 29	12345 * †	32154 * †	23154 †	23154 †	51234 * †	54321 *	53214	54321 *	15423	12345 *	54321 *	54321 *	34521	21354	12345 *
	bout 30	41235 †	45321 †	21543 * †	54123 †	21543 * †	54321 *	54321 *	54132	51234	34521	51432	51432	54321 *	12354	12345 *
	bout 31	21543 * †	43215 * †	23154 †	34521 †	21534 †	54312	54321 *	54132	54321 *	45123	15432	51432	53214	32154	23451
	bout 32	12345 * †	54321 * †	21543 * †	12354 †	51234 * †	54321 *	54321 *	54321 *	15234	41235	54321 *	54321 *	13254	15324	12345 *
	bout 33	45321 †	45132 †	54321 * †	12345 * †	45123 * †	54321 *	54321 *	12453	51432	12534	54321 *	51243	15432	54321 *	12345 *
	bout 34	12345 * †	43215 * †	21543 * †	23451 * †	54123 †	54321 *	54321 *	54321 *	12345 *	54321 *	51324	54321 *	54321 *	54321 *	23451
	bout 35	12345 * †	15432 †	23154 †	23451 * †	12354 †	54321 *	54321 *	15432	54321 *	54123	54321 *	51432	54321 *	54321 *	45123
	bout 36	43521 †	15432 †	21543 * †	34251 †	54123 †	54321 *	54312	13254	32145	43215	54321 *	54321 *	32154	12345 *	23451
	bout 37	51243 †	15432 †	21543 * †	12345 * †	51243 †	54312	54321 *	54321 *	54321 *	12345 *	54321 *	54321 *	54321 *	21543	51234
	bout 38	15432 †	32154 * †	32154 * †	34512 * †	54123 †	54312	34215	43215	23154	12345 *	54321 *	54321 *	23451	32415	23451
	bout 39	12354 †	43215 * †	12354 †	54123 †	45123 * †	54321 *	54123	12543	54321 *	52341	54321 *	54321 *	54321 *	54321 *	51234
	bout 40	32145 †	43215 * †	32154 * †	54123 †	45123 * †	54321 *	34512	52314	54321 *	43215	54321 *	15432	15432	21543	51432
Phase 2	bout 41	342	21543 *	21543 *	23451 * †	12345 *	12	54321 *	51234	1234	23451	54321 *	54321 *	53214	32154	12345 *
	bout 42	321	32154 *	54123	34512 *	32541 †	1235	54321 *	12543 †	51432	43215	54321 *	54312	32154	21534 †	45321
	bout 43	23451 *	45321	15432	23451 * †	34512 * †	54321 *	54321 *	51234	42135	41235	54321 *	54321 *	54321 *	34521	12345 *
	bout 44	43215 *	43215 *	23145	45123 *	423	152	54312	15432 †	52134	12345 *	54321 *	54321 *	54321 *	34521	45321
	bout 45	51234	54321 * †	15432	23451 * †	45123 *	54321 *	31524	54321 *	31524	43215	14325	54321 *	54321 *	43521	43125
	bout 46	21534	45132	21543 *	45123 *	32451 †	54312	54321 *	43215	54321 *	54321 * †	54321 *	54321 *	54321 *	34215	12345 *
	bout 47	54321 *	43215 *	42513 †	51432	12345 *	51432	54321 *	512	54321 *	12534	15432	54321 *	54321 *	23154 †	54321 *
	bout 48	12543	15432	45123 * †	34512 *	54123	23451	54321 *	54321 *	812	21354	54321 *	54321 *	54321 *	54321 *	12345 *
	bout 49	32451 †	34152	54132	43215 *	54231	54123	21345	541	43521	43215	54321 *	54321 *	51432	12345 *	23451
	bout 50	21345	23154	15432	32145	54123	45	32514	54321 *	54321 *	12543	4512	54321 *	32451	51432	12345 *
	bout 51	52134	43215 *	21543 *	23541 †	54123	51234	152	54321 *	21345	13452	54321 *	54321 *	54321 *	21543 †	54123 †
	bout 52	54321 *	54312 †	14325	43521	13	12345 * †	54321 *	54312	51234	21345	54321 *	54321 *	13245	54312	51234 †
	bout 53	51234	45213	54123	21543 * †	34512 * †	542	43512	15423 †	51234	51234 †	54321 *	54321 *	43215	12345 *	23451
	bout 54	21345	52143 †	32415	54213	21345	54321 *	54321 *	15432 †	15432 †	32145	13425	54321 *	13452	32154	45123
	bout 55	12345 *	54321 * †	43215 * †	43251	54123	54321 *	54321 *	15432 †	32154	54123 †	15432	54321 *	152	23154 †	54321 * †
	bout 56	53214	34125	12345 *	45123 *	43215 *	54312	21345	54321 *	54123	51234 †	54312	54321 *	15432	12354	45123
	bout 57	51243	51243 †	43215 * †	23451 * †	43	23451	15432 †	15432 †	12345 * †	23451	32145	54321 *	43152	54321 *	45123
	bout 58	51234	351	21534	12345 *	32514 †	21543	123	15432 †	54321	51234 †	12543	51432	13452	12354	43512
	bout 59	41532	12543	1345	12534	12345 *	54321 *	15432 †	15432 †	12435 †	12345 *	51432	54321 *	54321 *	25413 †	12345 *
	bout 60	52341	32154 *	43215 * †	123	32541 †	12345 * †	531	54312	54321 *	51234 †	54321 *	54321 *	51432	21543 †	45123
	bout 61	53214	21354	21543 *	23451 * †	45132	23451	12354 †	12543 †	123	51234 †	52341	54321 *	54321 *	54321 *	5134
	bout 62	32145 †	32154 *	12543	23451 * †	32514 †	54123	43521	15432 †	43215	51234 †	54321 *	54321 *	13254	12345 *	45123
	bout 63	21345	54321 * †	51432	23154 †	45123 *	54312	125	15432 †	15423 †	23154	54321 *	54321 *	15342	23415 †	54312
	bout 64	54321 *	15432	54321 *	12345 *	51234 *	54321 *	54321 *	15	54321 *	51432 †	54321 *	51432	15432	34512	54321 *
	bout 65	23154	21543 *	21543 *	23145 †	51234 *	21543	43215	21543	54321 *	51234 †	54321 *	54321 *	32154	54321 *	45123
	bout 66	53241	54321 * †	51243	23451 * †	12345 *	12345 * †	43215	51243	43215	51234 †	12345 *	21543 †	15432	12345 *	12345 *
	bout 67	32145 †	12543	1243	51234 *	34512 * †	54321 *	15432 †	12543 †	543	12345 *	54123	54321 *	12	12345 *	45123
	bout 68	32154 * †	21543 *	43215 * †	23451 * †	12354	15432 †	23451	54321 *	54321 *	51234 †	15432	54321 *	54321 *	45132	12345 *
	bout 69	32154 * †	12	15432	23451 * †	34512 * †	21345	43512	54321 *	54312	23451	54321 *	54321 *	54321 *	51234	234
	bout 70	34512 * †	52143 †	43215 * †	23145 †	31254 †	51	34512	13425 †	51234	51234 †	12345 *	54321 *	54321 *	21543 †	12345 *
	bout 71	12345 *	54321 * †	45321 †	51234 *	42351	15432 †	21534	541	15432 †	12345 *	45321	54321 *	15432	54123	54321 *
	bout 72	12345 *	35124	21543 *	45123 *	12345 *	54123	54312	51243	54312	12345 *	54321 *	54321 *	54321 *	45123	12345 *
	bout 73	34512 * †	12543	15432	23451 * †	34512 * †	12	12345 †	15432 †	15432 †	12345 *	12345 *	32154	54321 *	51234	12345 *
	bout 74	15432	32154 *	32154 *	54123	23451 *	15432 †	51342	15432 †	15432 †	12345 *	54321 *	21543 †	15432	51243	12345 *
	bout 75	34512 * †	54321 * †	54321 *	34512 *	51234 *	15432 †	23451	12	15432 †	21345	54321 *	54321 *	21543	21543 †	12345 *
	bout 76	32145 †	435	43215 * †	23451 * †	34512 * †	25431	35142	15234 †	53412	12345 *	12345 *	51432	12345 *	45123	45123
	bout 77	32154 * †	21543 *	43215 * †	45123 *	23451 *	12543 †	23154	51234	15432 †	13245	54321 *	51432	15432	51234	12345 *
	bout 78	12354	54321 * †	21543 *	23451 * †	34512 * †	15234 †	12534 †	15432 †	43215	51234 †	54321 *	51432	543	15432	12345 *
	bout 79	54231	21354	43215 * †	23451 * †	23451 *	15432 †	15432 †	15	43125	51432 †	54321 *	54321 *	45312	54321 *	12345 *
	bout 80	34512 * †	54321 * †	43215 * †	34512 *	23145	54321 *	53124	12543 †	12543 †	15432	54321 *	4512	43215	32154	15234

Trade-off between distance and prioritization of high-reward sites in traplining bumblebees

by Lihoreau, Chittka & Raine

Fig. S3. Flower visitation sequences (excluding revisits to the same flower). For each bee (columns 1-15), visitation sequences are sorted in chronological order (bouts 1-40: phase 1; bouts 41-80: phase 2). Numbers 1-5 in each row refer to the spatial location of each flower in the array (see Fig. 1): hence a bee moving clockwise round the pentagonal array in order starting with a visit to flower 1 would be recorded as '12345'. Colour codes (44 combinations of font and cell colours) indicate the sequences observed significantly more often than expected by chance during phases 1 and 2 (multinomial test with a random probability 0.0083: $p < 0.05$). *: sequences minimizing travel distance (phases 1 and 2); †: sequences maximizing initial rate of food intake (phase 2). Incomplete sequences, in which at least one of the five flowers was not visited, are indicated using a red font and white cell background (these sequences were not included in analyses).
