# Supplementary material for: Trade-off between travel distance and prioritization of high-reward sites in traplining bumblebees
Source: Funct Ecol. 2011 Dec;25(6):1284–92. doi: 10.1111/j.1365-2435.2011.01881.x (PMC3260656; doi:10.1111/j.1365-2435.2011.01881.x)
Supplement: Supplementary file 4 [file fec0025-1284-SD4.pdf]

Table S1. Cartesian coordinates (x; y, scale in metres) of the nest-box, the artificial flowers, and the landmarks in the flight room.

	x	y
Nest-box (experiment 1)	3.70	5.10
Nest-box (experiments 2 and 3)	3.70	0.10
Flower 1	1.65	2.28
Flower 2	0.38	6.17
Flower 3	3.70	8.59
Flower 4	7.02	6.18
Flower 5	5.75	2.28
Landmark a	1.00	0.00
Landmark b	1.00	8.70
Landmark c	6.30	8.70
Landmark d	6.30	0.00
