# Supplementary material for: Trade-off between travel distance and prioritization of high-reward sites in traplining bumblebees
Source: Funct Ecol. 2011 Dec;25(6):1284–92. doi: 10.1111/j.1365-2435.2011.01881.x (PMC3260656; doi:10.1111/j.1365-2435.2011.01881.x)
Supplement: Supplementary file 5 [file fec0025-1284-SD5.pdf]

Table S2. Minimum distances (in cm) the bees would travel if they visited each flower once in a clockwise or anticlockwise sequence, in relation to the first visited flower (see Fig. 1). *: shortest possible distance.

Flower visitation sequence		Flight distance (experiment 1)	Flight distance (experiments 2 and 3)
Clockwise	12345	2338 *	2238 *
	23451	2338 *	2631
	34512	2338 *	3181
	45123	2338 *	3181
	51234	2338 *	2631
Anticlockwise	15432	2338 *	2631
	21543	2338 *	3181
	32154	2338 *	3181
	43215	2338 *	2631
	54321	2338 *	2238 *
